# Real-World Safety and Effectiveness of Ganirelix for Ovarian Stimulation in Chinese Women: A Multicenter, Prospective, Single-Arm, Observational Study

**DOI:** 10.1177/26884844251387017

**Published:** 2025-10-09

**Authors:** Jia Li, Fei Gong, Lihong Geng, Xiaohong Wang, Zhaolian Wei, Jianqiao Liu, Yong Zeng, Jue Wang, Keith Gordon, Sisi Chen, Rui Yang, Rong Li

**Affiliations:** ^1^ Department of Obstetrics and Gynecology, Reproductive Medical Center, Peking University Third Hospital, Beijing, China.; ^2^ Clinical Research Center for Reproduction and Genetics in Hunan Province, Reproductive and Genetic Hospital of CITIC-XIANGYA, Changsha, China.; ^3^ Xinan Gynecological Hospital, Chengdu, China.; ^4^ Reproductive Medical Center, Tangdu Hospital, Air Force Medical University, Xi’an, China.; ^5^ Department of Obstetrics and Gynecology, The First Affiliated Hospital of Anhui Medical University, Hefei, China.; ^6^ Department of Obstetrics and Gynecology, Center for Reproductive Medicine, Guangdong Provincial Key Laboratory of Major Obstetric Diseases, The Third Affiliated Hospital of Guangzhou Medical University, Guangzhou, China.; ^7^ Shenzhen Key Laboratory of Reproductive Immunology for Peri-implantation, Shenzhen Zhongshan Institute for Reproduction and Genetics, Fertility Center, Shenzhen Zhongshan Urology Hospital, Shenzhen, China.; ^8^ MA & OR, Organon Research and Development, Organon China, Shanghai, China.; ^9^ MA & OR, Organon Research and Development, Organon & Co., Jersey City, New Jersey, USA.

**Keywords:** ganirelix, gonadotropin-releasing hormone antagonist, ovarian stimulation, safety

## Abstract

**Background:**

This study aimed to evaluate the safety and effectiveness of ganirelix acetate for Chinese women undergoing ovarian stimulation (OS) in real-world clinical practice.

**Methods:**

This multicenter (16), prospective, single-arm, observational, post-authorization safety study included 1025 Chinese women receiving at least one dose of ganirelix during OS. Safety endpoints were collected until 5 weeks after embryo transfer. The investigator assessed the causality and seriousness of an adverse event (AE). Effectiveness and neonatal outcomes were collected from medical records and phone call follow-ups. The study had a probability of >95% to observe an AE occurring at 0.3% with a sample size of 1000.

**Results:**

The occurrence of overall AEs, drug-related AEs, and serious AEs (SAEs) was 12.1% (124/1025), 1.3% (13/1025), and 2.4% (25/1025), respectively. None of the SAEs were drug related, according to the investigator. Two (0.2%) patients discontinued treatment due to an AE. The most reported AE was ovarian hyperstimulation syndrome (4.8%, 49/1025), among which 21 cases were reported as SAE. The proportion of patients with a premature luteinizing hormone (LH) rise >10 IU/L) was 2.5% (26/1025), and one patient had a premature ovulation. The live birth rate was 34.9% (358/1025) per start cycle with a cumulative live birth rate of 42.1% (432/1025). Neonatal malformations occurred in 1.3% (7/523) of the neonates.

**Conclusions:**

This study indicates that the safety profile and effectiveness of ganirelix were clinically acceptable, and no new safety signals emerged in real-world clinical practice for Chinese women with OS. The study was registered on Chinadrugtrial.org (identifier: CTR20150284) (http://www.chinadrugtrials.org.cn) and ENCePP.eu (identifier: EUPAS8737) (https://catalogues.ema.europa.eu).

## Background

Infertility is considered the fifth most severe global disability according to the World Health Organization, and the estimated global infertility prevalence for 2022 revealed that 16.66% of individuals experience infertility at some juncture.^1^ Encouragingly, the probability of parenthood is improved with the advancement of medical assisted reproductive (MAR), such as *in vitro* fertilization (IVF) and intracytoplasmic sperm injection (ICSI). One of the core procedures of IVF/ICSI is ovarian stimulation (OS), which is required to control premature luteinizing hormone (LH) surges, ovulation, and luteinization during multiple follicular growths. In the early 1980s, the gonadotropin-releasing hormone (GnRH) agonists (GnRH-a) were applied in either long or short (flare-up) protocols. Since 1999, GnRH antagonists (GnRH-ant) have been introduced; with their mode of action competitively binding to the GnRH receptor, they provide immediate but reversible endogenous LH suppression that allows a short and straightforward treatment regimen for IVF patients.^2^

Application of a GnRH-ant protocol in OS achieves comparable reproductive outcomes with a shorter gonadotropin (Gn) duration compared with the long GnRH-a protocol,^3,4^ which is considered to improve patient convenience and compliance. Furthermore, GnRH-ant was associated with a significantly lower incidence of severe ovarian hyperstimulation syndrome (OHSS) than a long GnRH-a protocol.^5–7^ Thus, the GnRH-ant protocol has become the gold standard in clinical practice worldwide.

To date, two GnRH-ant (ganirelix and cetrorelix) have been approved for OS in China. Ganirelix was the first antagonist approved in the United States in July 1999 and was subsequently approved in China in June 2013. Large randomized efficacy and safety trials, including infant follow-up, provided solid evidence of the efficacy and safety of ganirelix treatment in comparison to various long GnRH-a, including buserelin, triptorelin, and leuprorelin.^3,8,9^ The registration clinical trial of ganirelix in China compared ganirelix treatment to a long triptorelin protocol and enrolled 233 women aged 35 or below with a good IVF prognosis.^4^ Ongoing pregnancy rates per start cycle were 39.8% (ganirelix) and 39.2% (triptorelin). Although both treatments were well tolerated, cancellation due to the risk of OHSS was less frequent with ganirelix (1.8%) than with triptorelin (7.5%), as in previous trials, less follicle-stimulating hormone (FSH) was needed in the ganirelix protocol than in the long GnRH-a protocol.

Thereafter, several other studies were conducted in broader Chinese populations with a major focus on the outcome of OHSS, which was of particular interest in the Chinese IVF population, as they are younger and leaner than those outside Asia.^10,11^ With respect to the safety of women who have undergone IVF and their offspring, a large prospective pregnancy and infant follow-up was performed comparing the obstetrical and neonatal data (839 pregnancies and 969 fetuses) following ganirelix treatment with a historical cohort (753 pregnancies and 963 newborns) following long GnRH-a treatment. The study indicated that the occurrence rate of significant congenital malformations in fetuses was similar between the ganirelix (5.0%) and the GnRH-a (5.4%) groups.^12^

However, the safety of women who have undergone IVF and their offspring in real-world practice in China has not been reported in the literature. Therefore, the current multicenter, prospective, single-arm, observational study aimed to evaluate the safety, including neonatal health conditions, and effectiveness of ganirelix in Chinese women with IVF procedures in real-world clinical practice. This study will provide additional evidence on the safety and effectiveness of ganirelix in real-world use in China and serve as a post-authorization safety study (PASS) since its approval in the country.

## Methods

### Study design and setting

This was a prospective, multicenter, single-arm, observational PASS conducted in China. Sixteen IVF centers were included to represent real-world practice. The investigators, who specialized in IVF, screened and enrolled the subjects to collect data from their medical records of routine clinical practice. Subject enrollment and data collection began on April 28, 2015 (first subject first visit), and ended on June 6, 2017 (last subject last visit).

The study was codesigned by the sponsor and the leading principal investigator (PI) (R.L.). The sponsor performed statistical analyses, and the leading PI and the team (J.L., R.Y., and R.L.) analyzed and interpreted the study results clinically. The study complied with local laws and regulations and followed China’s National Medical Products Administration guidelines and regulations. The study protocol was approved by the Independent Ethics Committee of each site before the start of the study. All women provided written informed consent before commencing any study-specific procedure and data collection. The study was registered on Chinadrugtrial.org (identifier: CTR20150284) (http://www.chinadrugtrials.org.cn) and ENCePP.eu (identifier: EUPAS8737) (https://catalogues.ema.europa.eu).

### Participants

Chinese women who signed the informed consent form, accessed OS, were deemed suitable for a GnRH-ant protocol assessed by reproductive endocrinologists, and had completed at least one dose of ganirelix were eligible for inclusion in this study. This PASS did not set specific exclusion criteria as it collected and evaluated safety data in real-world clinical settings.

### Study procedures and data collection

IVF treating physicians determined OS regimens based on their experiences and preferences as the first step before patient assessment. Women who were stimulated with Gn and ganirelix had oocyte retrievals after triggering, followed by conventional IVF or ICSI. Pregnancy tests and the ultrasound were performed 2 and 5 weeks after fresh embryo transfer (fET) or frozen–thawed embryo transfer (FET). All treatment was based on the treating physicians’ judgment and routine clinical practice. Subjects were deemed to have completed the protocol-required procedures and data collection if they obtained oocytes, had at least one ET during the study, and provided final pregnancy outcomes during telephone follow-up. Safety and medical events were documented by the treating physicians, who examined the patient routinely, during every on-site clinic visit or phone call follow-up during ganirelix treatment and up to 5 weeks after ET. Then, the investigator assessed independently if the event was an adverse event (AE) and confirmed the causality and severity. Effectiveness data were collected by the investigator’s medical chart review based on the medical records at each site. Neonatal data were collected by the investigator *via* follow-up phone calls (Supplementary Fig. S1).

### Outcome assessments

Safety endpoints included overall AEs, drug-related AEs, serious AEs (SAEs), and discontinuation due to an AE. AE was defined as any untoward medical occurrence in a participant who was administered the investigational product, irrespective of suspected causal relationship. A drug-related AE was confirmed if there was a reasonable temporal correlation and causation between AE and ganirelix. An SAE was defined as an occurrence leading to death, life-threatening, permanent, or serious disability/incapacity, resulting in hospitalization or prolonged hospitalization, and congenital anomalies/birth defects or cancer. SAE classification was determined solely by predefined outcome criteria, irrespective of intensity grading.

Overdosing and a medical event deemed significant by the investigator were also reported as an SAE. The frequency and intensity of OHSS were assessed at the treating physician’s discretion and finally confirmed by the investigator according to the standards used in routine clinical practice.

Effectiveness endpoints included LH rise (defined as a serum LH >10 IU/L), premature ovulation (clinically judged by experienced physicians in routine clinical practice), number of oocytes retrieved, number of embryos, clinical pregnancy rate, and live birth rate. Neonatal endpoints included neonatal weight, gestational age at delivery, mode of delivery, pregnancy type (single or multiple), and the occurrence of congenital abnormalities.

### Data analysis

The primary analysis was based on safety endpoints and summarized as count, point estimate, and corresponding 95% confidence interval calculated by the Wilson score method. Demographic and clinical data, including neonatal outcomes, were analyzed using descriptive statistics. Continuous variables were expressed as mean (standard deviation [±SD]) and median (minimum, maximum). Categorical variables were expressed as frequencies or proportions.

The safety analysis set included the all subjects as treated (ASaT) population defined as all women who received at least one dose of ganirelix. The effectiveness analysis was also conducted in the ASaT population but presented separately as per OS attempt (all women attempting OS treatment) and per ET attempt (all women reached embryo transfer [ET] stage). The neonatal analysis was conducted in all live-born neonates. All analyses were performed using SAS without statistical or clinical imputation for missing data.

### Sample size and power calculation

Based on the ganirelix registration study in China,^4^ the proportion of ganirelix-related AEs and total SAE was 0.9% and 3.6%, respectively. With an approximate sample size of 1000 women, there was a 95% probability of observing: (1) at least one event with an incidence rate ≥0.3%; (2) a minimum of five events with an incidence rate of ≥1%; and (3) at least 39 cases with an incidence rate of ≥5%.

## Results

### Participants’ disposition, baseline, and OS characteristics

A total of 1025 women received at least one dose of ganirelix were included in the study (ASaT); 1008 (98.3%) women had oocyte retrieval, 929 (90.6%) women obtained transferable embryo(s), and 849 (82.8%) women had at least one ET (Supplementary Fig. S2). One hundred and nineteen (11.6%) women discontinued the study, and the top 3 reasons for discontinuations were (1) did not perform the FET cycle before the study was completed (34 women); (2) started a new OS cycle (24 women); and (3) insufficient ovarian response (17 women) (Supplementary Table S1). Two women discontinued the study because of an AE (one itching and one dermatitis).

The participant’s demographics and OS characteristics were summarized in Table 1. The mean age was 32.2 years, the mean weight was 57.1 kg, and the mean body mass index was 22.3 kg/m^2^. In the ASaT set, 22 (2.15%) women had a ganirelix dose lower than 0.25 mg, and overdose of ganirelix was seen in 12 (1.2%) women.

**Table 1.** Demographic and Ovarian Stimulation Characteristics of the Study Patients (*N* = 1025)

	Value
Demographic	
Age, mean (SD), years	32.2 (5.3)
Weight, mean (SD), kg	57.1 (9.2)
BMI, mean (SD), kg/m^2^	22.3 (3.2)
OHSS history,^a^ *n* (%)	101 (9.9%)
OS characteristics	
Duration of FSH stimulation, mean (SD), days	9.8 (1.9)
Total FSH dose, mean (SD), IU	2140.8 (878.7)
Duration of ganirelix, median (range), days	5.0 (1.0–13.0)
Total ganirelix dose, median (range), mg	1.25 (0.25–3.25)
Ganirelix underdose, *n* (%)	22 (2.1%)
Ganirelix overdose, *n* (%)	12 (1.2%)
LH rise,^b^ *n* (%)	26 (2.5%)
Before ganirelix	8 (0.8%)
During ganirelix	18 (1.8%)
Premature ovulation, *n* (%)	1 (0.1%)

^a^ OHSS history refers to the enrolled patients who have experienced OHSS before the start of the present study.

^b^ LH rise was defined as serum LH >10 IU/L during OS.

BMI, body mass index; FSH, follicle-stimulating hormone; LH, luteinizing hormone; OS, ovarian stimulation; SD, standard deviation.

### Safety and tolerability

Of all the 1025 women in the safety set, 124 (12.1%) experienced at least one AE during the treatment period, and the prevalence of AEs, drug-related AEs, SAEs, and AEs leading to discontinuation was low (Table 2). The most frequently reported AEs were OHSS (49 [4.8%]), among which 13 (1.3%) were clinically assessed as severe OHSS. Twenty-five (2.4%) SAEs were reported, predominantly OHSS (21 cases), including events of mild to moderate clinical severity requiring hospitalization. The majority of the SAEs were mild to moderate in severity (Supplementary Table S2). The investigators determined that all SAEs were unrelated to the study drug and were resolved during the study. No death was reported or occurred during the treatment period (Table 2).

**Table 2.** Adverse Events in the Treated Sets (All Participants as Treated, *N* = 1025), Presented as *n* (%)

Variables	*n* (%)
≥1 AE	124 (12.1%)
SAEs^a^	25 (2.4%)
Drug-related AEs^b^	13 (1.3%)
Discontinued due to AEs	2 (0.2%)
Drug-related AEs^b^	2 (0.2%)
SAEs^a^	0 (0%)
AEs occurring in ≥0.3% of patients	
OHSS	49 (4.8%)
Mild	17 (1.7%)
Moderate	19 (1.8%)
Severe	13 (1.3%)
Intentional drug overdose	14 (1.4%)
Bloating	11 (1.1%)
Virginal discharge	7 (0.7%)
Abdominal pain	4 (0.4%)
Nausea	3 (0.3%)

^a^ None of the SAEs were considered drug-related AEs, and no deaths were reported.

^b^ Determined by the investigator to have been related to ganirelix.

AE, adverse events; OHSS, ovarian hyperstimulation syndrome; SAE, serious adverse events.

There was no drug-related SAE reported during the treatment period, and 13 (1.3%) women experienced 14 drug-related AEs, of which intentional drug overdose by the physicians (4 cases) was the most reported AE. Two patients (0.2%) discontinued the study due to itching and dermatitis, respectively, and both AEs were mild in severity and fully recovered after discontinuing ganirelix (Supplementary Table S2).

### LH rise and clinical outcomes

LH rise (>10 IU/L) was observed in 26 (2.5%) patients during OS, of which 18 (1.8%) cases were reported after ganirelix initiation (Table 1). The investigator deemed only one patient to have premature ovulation and reported it as an AE.

The mean number (SD) of retrieved oocytes and embryos was 11.2 (8.0) and 6.2 (6.0), respectively. After the first ET, the clinical pregnancy rate per start cycle was 40.6%, with a live birth rate of 34.9%. The cumulative clinical pregnancy rate after multiple ETs per start cycle was 42.1%, with a cumulative live birth rate of 39.4% (Table 3).

**Table 3.** Clinical Outcomes (All Participants as Treated, *N* = 1025)

Characteristics	Mean (SD)/*n* (%)
Number of oocytes retrieved	11.2 (8.0)
Number of embryos	6.2 (6.0)
Number of transferred embryos	1.9 (0.4)
First ET,^a^ per start cycle^b^	
hCG (+)	458 (44.7%)
Clinical pregnancy rate	416 (40.6%)
Live birth rate	358 (34.9%)
First ET,^a^ per transfer cycle (*N* = 849^c^)	
hCG (+)	458 (53.9%)
Clinical pregnancy rate	416 (49.0%)
Live birth rate	358 (42.2%)
Multiple ET,^d^ per start cycle^b^	
Cumulative clinical pregnancy rate	432 (42.1%)
Cumulative live birth rate	404 (39.4%)

^a^ Pregnancy outcome after first fresh or frozen ET.

^b^ Patients with controlled ovarian stimulation.

^c^ Patients with at least one ET cycle.

^d^ Pregnancy outcome after multiple ET in the study period.

ET, embryo transfer; hCG, human chorionic gonadotropin.

### Neonatal outcomes

Table 4 presents the neonatal characteristics of 523 infants. Four hundred (76.5%) infants were delivered by caesarean section, and 26 (5%) infants had preterm birth. Congenital malformations were observed in 7 (1.3%) fetuses with 11 abnormalities. All 11 abnormalities were commonly seen in clinical practice, and none was related to ganirelix judged by investigators.

**Table 4.** Neonatal Outcomes of Infants (*N* = 523)

	Mean (SD)/*n* (%)
Livebirth (*N* = 404)^a^	
Singleton	286 (70.8%)
Twins	117 (29.0%)
Triplets	1 (0.2%)
Delivery characteristics	
Spontaneous vaginal	55 (10.5%)
Induced vaginal	39 (7.5%)
Caesarean section	400 (76.5%)
Forceps	2 (0.4%)
Not reported	27 (5.2%)
Gestational age (weeks)	36.9 (2.7)
Preterm birth	26 (5.0%)
Birth weight (g)	2966.5 (742.5)
Congenital abnormalities	7 (1.3%)
Congenital abnormalities related to ganirelix	0 (0%)
Type of congenital abnormalities	
Congenital heart disease	3 (0.6%)
Polydactyly	1 (0.2%)
Hand abnormalities	2 (0.4%)
Foot abnormalities	2 (0.4%)
Cleft lip	1 (0.2%)
Volvulus malformation	1 (0.2%)
Cytogenetic abnormalities	1 (0.2%)

^a^ Number of women with live infant(s).

## Discussion

This PASS further supports the application of ganirelix in the Chinese population in real-world clinical practice. The safety profile and effectiveness of ganirelix were clinically acceptable with no new safety signals emerging. Meanwhile, ganirelix is an effective treatment for inhibiting premature ovulation during OS, with favorable pregnancy and neonatal outcomes.

The most frequently reported AE in this study was OHSS (4.8%), a well-known pharmacological effect of FSH rather than GnRH-ant treatment. This incidence is similar to the incidence of OHSS (4.5%) in the previous ganirelix registration trial in China.^4^ The further minimization of OHSS incidences is of the most interest in China, as the patients are generally younger and have a lower body weight than those in the United States and Europe. In the past decade, GnRH-ant treatment has been further evaluated owing to the use of GnRH-a for triggering instead of human chorionic gonadotropin (hCG). This approach, followed by the freezing of all embryos (“freeze-all”), reduced the risk of developing OHSS induced either by exogenous hCG or endogenous hCG.^13,14^ Similar to the observations in other countries, the GnRH-ant protocol has lowered OHSS incidences in China, especially in high responders. The ganirelix-related AEs accounted for 1.4% in this study, and the most frequently reported AEs were intentional drug overdoses. Besides, 0.5% of women had a local tolerance reaction, consistent with the ganirelix dose-finding study^15^ with a severe local tolerance reaction of 1.2%. It should be noted that the investigators proactively collected the safety data in this study. They enquired about each new AE during every site visit and phone call follow-up. Therefore, we believe the AEs in this study were more exhaustively reported compared to those reported by patients themselves.

The efficacy of ganirelix in preventing premature LH surges has been well-established in previous trials, and a pooled analysis indicated that the incidence of LH rises (LH >10 IU/L) before ganirelix treatment was 6.6% on ganirelix start day 6.^16^ In general, women with early LH rises at stimulation day 6 are high responders but with similar ongoing pregnancy rates following fET compared to women without an early LH rise.^16,17^ In contrast, women with a late LH rise during ganirelix treatment, which was reported with an incidence of 2.3%, may have a reduced chance of ongoing pregnancy, especially when LH rise is coupled with a premature progesterone rise. The total incidence of premature LH rises during ganirelix treatment in the present study was 1.8%, with only one case determined by the researcher as early ovulation. In addition, it was noted that four patients received a higher daily dose than 0.25 mg ganirelix to reduce the risk of a premature LH surge, which is apparent in low responders^18^ and in patients who are not compliant with daily antagonist injection.^15^ Based on previous studies, the incidence of premature LH rises among women who did not take GnRH-ant was up to 38%.^19^

In the present study, the live birth rate per started cycle was 34.9%, consistent with a previous Chinese study that reported a rate of 37.5% for live births in the ganirelix group.^20^ Furthermore, a Chinese national report indicated a pregnancy rate of 29.2% per aspiration and 52.1% per transfer, which were also consistent with the study with a clinical pregnancy rate of 49.0% per transfer.^21^ The consistent outcomes across studies suggest that ganirelix offers a reliable and effective treatment option in assisted reproductive technology. In regard to birth outcome, note that 11 neonatal abnormalities were reported in 7 (1.3%, *N* = 523) newborns, and none was related to ganirelix according to investigators’ medical judgment. This incidence was comparable to the birth defect rate in the Chinese population (1.35%)^22^ and in neonates with MAR (1.64%).^23^ Besides, this incidence was similar to the study of cetrorelix, which showed that the malformation rate among all live births, stillbirths, and aborted fetuses was 3.1%.^24^ Overall, the current study’s findings indicated that the use of ganirelix had no noticeable impact on neonatal anomalies.

However, the current study had several limitations. First, this study was a single-arm observational study without a control group, which limits our ability to make direct comparisons and draw definitive conclusions about the effectiveness of the intervention. Future studies should consider incorporating a control group to provide more robust evidence and enhance the generalizability of the findings. Second, this study’s sampling and consent process might introduce selection bias. The sampling women treated with ganirelix were chosen by physicians’ experiences and preferences; neither random sampling nor continuous sampling was applied. Third, the study was conducted between April 2015 and June 2017. As clinical practice evolves, the safety and effectiveness results must be interpreted based on the current clinical situation.

## Conclusions

This observational safety study included 1025 Chinese women treated in routine practice in 16 Chinese IVF centers and confirmed previous findings in registration trials. As the study was conducted in a routine clinical setting at multiple IVF centers, the effectiveness results of ganirelix were more reflective of the real world. Finally, the study provided evidence for clinical decision making as it presented safety versus effectiveness profiles simultaneously. In conclusion, this real-world PASS showed that the safety profile and effectiveness of ganirelix treatment were consistent with those reported in the clinical development program and published literature. No new safety signal emerged in this study, and the occurrence of neonatal malformations was low. Ganirelix is a suitable GnRH-ant to be used in Chinese women undergoing MAR.

## Supplementary Material

Supplementary Figures

Supplementary Tables

## Data Availability

Study data may be shared upon external request to the leading PI and the sponsor.

## Abbreviations Used

AE: adverse event
ASaT: all subjects as treated
ET: embryo transfer
fET: fresh embryo transfer
FET: frozen–thawed embryo transfer
FSH: follicle-stimulating hormone
GnRH-a: gonadotropin-releasing hormone agonists
GnRH-ant: gonadotropin-releasing hormone antagonists
ICSI: intracytoplasmic sperm injection
IVF: *in vitro* fertilization
LH: luteinizing hormone
MAR: medical assisted reproductive
OHSS: ovarian hyperstimulation syndrome
OS: ovarian stimulation
PASS: post-authorization safety study
SAEs: serious adverse event
