# Supplementary material for: Real-World Safety and Effectiveness of Ganirelix for Ovarian Stimulation in Chinese Women: A Multicenter, Prospective, Single-Arm, Observational Study
Source: Womens Health Rep (New Rochelle). 2025 Oct 9;6(1):1119–26. doi: 10.1177/26884844251387017 (PMC12549165; doi:10.1177/26884844251387017)
Supplement: Supplementary Figures [file 26884844251387017_supplementary_figures.docx]

**
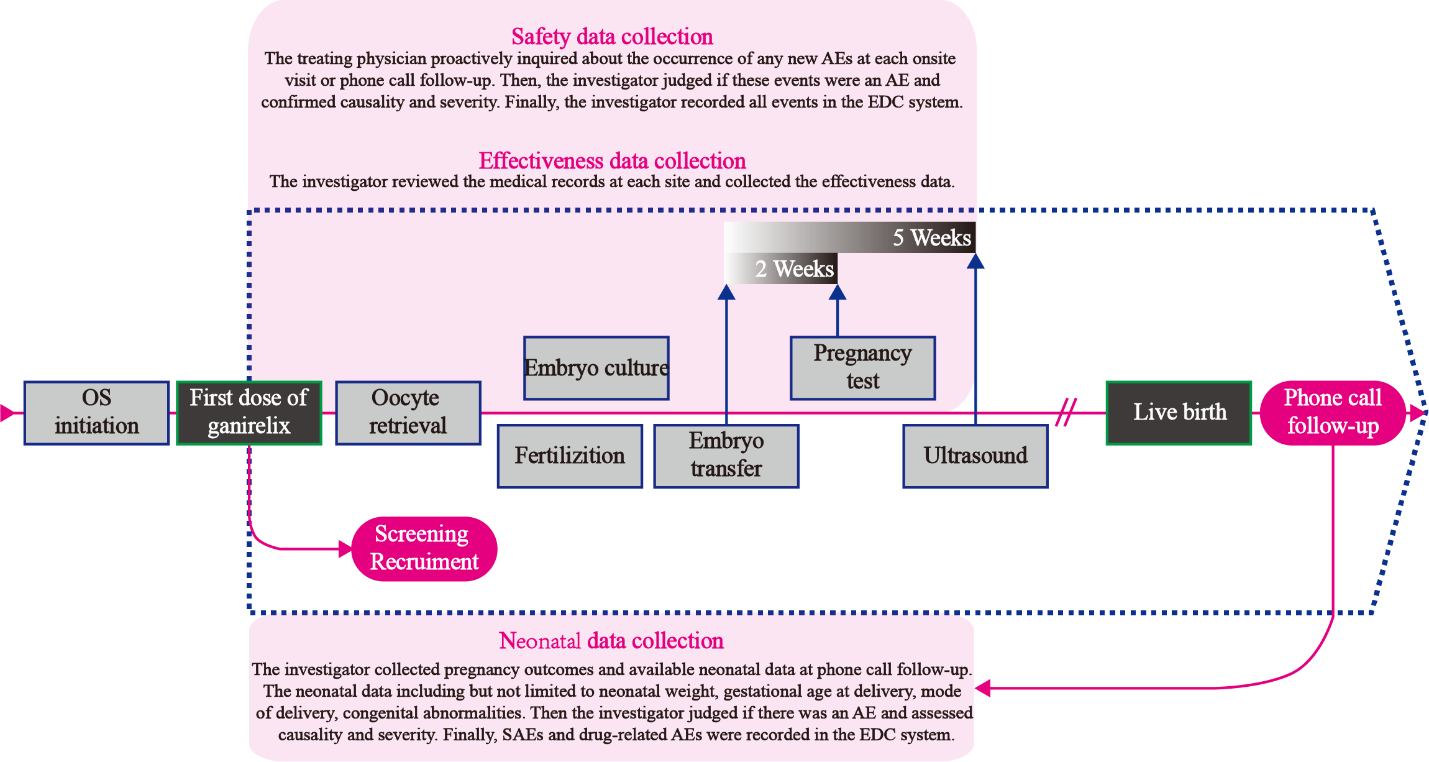
Fig S1** Flowchart of study procedures and data collection





**Fig S2** Total numbers of patients in each stage of IVF/ICSI

Abbreviations: PN prokaryotic nucleus, ET embryo transfer, IVF in vitro fertilization, ICSI Intracytoplasmic sperm injection
