# Supplementary material for: Real-World Safety and Effectiveness of Ganirelix for Ovarian Stimulation in Chinese Women: A Multicenter, Prospective, Single-Arm, Observational Study
Source: Womens Health Rep (New Rochelle). 2025 Oct 9;6(1):1119–26. doi: 10.1177/26884844251387017 (PMC12549165; doi:10.1177/26884844251387017)
Supplement: Supplementary Tables [file 26884844251387017_supplementary_tables.docx]

**Table S1.** Reasons for patients discontinued from the study

| Reasons | n (%) |
| --- | --- |
| Did not perform FET cycle before the study was completed | 34 (3.3%) |
| Started a new OS cycle | 24 (2.3%) |
| Insufficient ovarian response | 17 (1.7%) |
| Unfertilized or abnormal fertilization | 10 (1.0%) |
| No transferable embryo | 8 (0.8%) |
| Patients decided to withdraw | 7 (0.7%) |
| Transferred embryos were partially or totally from other OS cycles | 6 (0.6%) |
| Lost follow-up | 6 (0.6%) |
| Risk of OHSS | 4 (0.4%) |
| AE | 2 (0.2%) |
| Oocyte cryopreservation due to no sperm | 1 (0.1%) |
| **Total** | **119 (11.6%)** |

Abbreviations: FET frozen embryo transfer, OS ovarian stimulation, OHSS ovarian hyperstimulation syndrome, AE adverse events.

**Table S2.** Serious adverse events and drug-related AEs in the treated sets (all participants as treated, N = 1,025).

| Variables | n (%) | Severity |
| --- | --- | --- |
| **SAEs** | **25 (**2.4%) | NA |
| OHSS | 21 (2.0%) | Mild 10; Moderate 1; Severe 10 |
| Fallopian tube hydrocele | 1 (0.1%) | Moderate |
| Pain after oocyte retrieval surgery | 1 (0.1%) | Moderate |
| ovarian torsion | 1 (0.1%) | Moderate |
| adverse reactions to anesthesia | 1 (0.1%) | Mild |
| **Drug-related AEs^*^** | **14 (**1.4%) | NA |
| Intentional drug overdose | 4 (0.4%) | NA |
| Progesterone rise | 2 (0.2%) | Mild |
| Pruritus | 2 (0.2%) | Mild |
| Dermatitis | 1 (0.1%) | Mild |
| Dermatitis allergic | 1 (0.1%) | Mild |
| Injection site erythema | 1 (0.1%) | Mild |
| Injection site pain | 1 (0.1%) | Mild |
| Insomnia | 1 (0.1%) | Mild |
| Premature Ovulation | 1 (0.1%) | Mild |

^∗^Determined by the investigator to be related to the drug.

Abbreviations: AE adverse events, SAE serious adverse events, OHSS ovarian hyperstimulation syndrome, NA not applicable.
